# The Emerging Roles for Telomerase in the Central Nervous System

**DOI:** 10.3389/fnmol.2018.00160

**Published:** 2018-05-16

**Authors:** Meng-Ying Liu, Ashley Nemes, Qi-Gang Zhou

**Affiliations:** ^1^Department of Clinical Pharmacology, Pharmacy College, Nanjing Medical University, Nanjing, China; ^2^The Affiliated Hospital of Nanjing University Medical School, Nanjing Drum Tower Hospital, Nanjing, China; ^3^Department of Stem Cell Biology and Regenerative Medicine, Lerner Research Institute, Cleveland Clinic, Cleveland, OH, United States

**Keywords:** telomerase, central nervous system, proliferation, differentiation, apoptosis

## Abstract

Telomerase, a specialized ribonucleoprotein enzyme complex, maintains telomere length at the 3′ end of chromosomes, and functions importantly in stem cells, cancer and aging. Telomerase exists in neural stem cells (NSCs) and neural progenitor cells (NPCs), at a high level in the developing and adult brains of humans and rodents. Increasing studies have demonstrated that telomerase in NSCs/NPCs plays important roles in cell proliferation, neuronal differentiation, neuronal survival and neuritogenesis. In addition, recent works have shown that telomerase reverse transcriptase (TERT) can protect newborn neurons from apoptosis and excitotoxicity. However, to date, the link between telomerase and diseases in the central nervous system (CNS) is not well reviewed. Here, we analyze the evidence and summarize the important roles of telomerase in the CNS. Understanding the roles of telomerase in the nervous system is not only important to gain further insight into the process of the neural cell life cycle but would also provide novel therapeutic applications in CNS diseases such as neurodegenerative condition, mood disorders, aging and other ailments.

## Introduction

Telomeres are simple repeat sequences at the physical 3′ end of chromosomes (TTAGGG for human and mouse; Greider, 1996). Since 1989 when it was discovered that the maintenance of telomeric length was mediated by telomerase (Blackburn et al., 1989; Greider and Blackburn, 1989), a large number of studies have focused on the function of telomerase in stem cells. The structure of telomerase was revealed as a specialized ribonucleoprotein complex, consisting of a protein component telomerase reverse transcriptase (TERT) that serves as catalytic subunit (Counter et al., 1997; Harrington et al., 1997; Meyerson et al., 1997; Nakamura et al., 1997; Greenberg et al., 1998), and an essential telomerase RNA component (also known as TERC or RT). The TERC serves as a template for the elongation of a telomere catalyzed by TERT (Blackburn, 2001).

Neural stem cells (NSCs), like other types of stem cells, are self-renewing and multipotent (Gage, 2000). In the adult rodent brain, NSCs are mainly present in two specific regions: the subgranular zone (SGZ) in the dentate gyrus (DG) of the hippocampus and the subventricular zone (SVZ) of the lateral ventricles. In the SGZ, new dentate granule cells are produced from NSCs daily. In the SVZ, new neurons are also born every day and migrate through the rostral migratory stream (RMS) to the olfactory bulb to become interneurons (Gage, 2000; Goh et al., 2003; Alvarez-Buylla and Lim, 2004; Ma et al., 2005; Zhao et al., 2008; Ming and Song, 2011). TERT is present at high levels in NSCs and NPCs in the developing brain (Klapper et al., 2001; Cai et al., 2002) and adult (Caporaso et al., 2003), but then declines rapidly when stem/progenitor cells differentiate or die. Moreover, telomerase has an influence on several steps of the cell life cycle including proliferation, differentiation, survival, development and apoptosis (Mattson et al., 2008; Ferrón et al., 2009). Studies also show that telomerase has been closely associated with NSCs-/NPCs-related diseases in the central nervous system (CNS) including brain tumorigenesis (Kheirollahi et al., 2013), ischemia (Zhao et al., 2010; Li et al., 2011), neurodegenerative illness (Franco et al., 2006), affective disorders (Lee et al., 2010; Zhou et al., 2011) and schizophrenia (Kao et al., 2008; Porton et al., 2008). Here we will review published literature focusing on the relationship between telomerase, NSCs and other nerve cells in the CNS, as well as cellular proliferation, differentiation, survival, development, apoptosis and their applications and emerging relevance to CNS diseases.

## Structure and Function

The telomerase is composed of TERT and TERC in all species (Feng et al., 1995; Harrington et al., 1997). TERC acts as a template for the addition of TTAGGG at the end of telomeric DNA in humans (Feng et al., 1995; Harrington et al., 1997). Telomerase expression and activity is repressed in normal human somatic cells in adulthood (Meyerson et al., 1997; Masutomi et al., 2003). In contrast, it actives only in a small subset of adult cells, including stem cells and progenitor cells in renewal tissues, germline cells, mesenchymal stem cells and activated lymphocytes (Broccoli et al., 1995; Chiu et al., 1996; Wright et al., 1996; Martens et al., 2002; Serakinci et al., 2008). Accumulating evidence indicates that telomere shortening represents a marker and a mechanism of biological aging (Aubert and Lansdorp, 2008). Telomerase is implicated in this process as it contributes to DNA damage accumulation in ASCs (Epel, 2009; Wang et al., 2009; Sahin and Depinho, 2010). Moreover, mutational analysis and knockdown experiments showed that telomerase deficiency led to telomere loss and uncapping, causing progressive atrophy of renewal tissues, gradual depletion of stem cells and eventual failure of organ systems (Jaskelioff et al., 2011; Bär and Blasco, 2016). Above all, telomerase may play a critical role in cellular and organismal aging, and could be a potential target for anti-aging therapy (Bernardes de Jesus et al., 2012; Shay, 2016).

After numerous cycles of cell division, stem/progenitor cells in the adult body lacking telomerase stop proliferating and enter into a state of growth arrest called replicative senescence (Newbold, 1997). However, elevated telomerase expression is almost universal in human tumors which make it a hallmark of cancer cells (Kim et al., 1994). As telomerase counteracts telomere shortening during rounds of cellular proliferation by synthesizing new repeats (TTAGGG) for maintaining the telomeric length at the chromosomal termini, cancer cells are able to maintain their telomere to proliferate continuously without limits, making telomerase a promising target for cancer treatment (Collins and Mitchell, 2002). Accordingly, several telomerase-based strategies have been developed by scientists for cancer therapy and many are in advanced clinical trials (Kim et al., 1994; Vonderheide, 2002; Shay and Keith, 2008; Liu et al., 2010).

## Species Difference

Although the telomere is composed of TTAGGG repeats in all vertebrates, the length differs significantly in different species (Calado and Dumitriu, 2013). On average, the telomere in humans is about 5–15 kb long, while much longer telomere (50–100 kb) is detected in laboratorial mice (Calado and Dumitriu, 2013). Consistently, in marked contrast to humans, mice have higher levels of telomerase expression and activity (Prowse and Greider, 1995; Burger et al., 1997; Wright and Shay, 2000). The expression of telomerase declines dramatically upon differentiation of stem cells in humans; therefore, it is difficult to detect telomerase in somatic human cells. However, robust levels of telomerase activity are detected in a wide range of somatic tissues and cells in mice (Horikawa et al., 2005).

Horikawa et al. (2005) found that the activity of the mouse TERT promoter is 5.4–16 folds higher than that of the human TERT promoter, in which a non-conserved GC-Box functions critically. Studies investigating site-directed mutations revealed that a GC-box (CCCCGCCC) located at −31 to −24 and a putative E2F site (GCGCG) located at −13 to −9 in the human TERT promoter contribute to the repression of the activity of the human TERT promoter (Horikawa et al., 2005). Nonetheless, the molecular mechanisms of these differences remain largely obscure and need more research focus. The species difference of telomerase implies that it requires a humanized mouse model for deeply studying the role of telomerase in aging and cancer and more attention should be paid when studying the function of telomerase in the brain using regular laboratorial mice.

## Expression of Telomerase in the CNS

Telomerase highly exists in the brain at embryonic stages and declines gradually after birth except in adult stem cells (ASCs; Wright et al., 1996; Greenberg et al., 1998; Martín-Rivera et al., 1998; Armstrong et al., 2005). Particularly, TERT expression and activity are confirmed in adult brain tissues including the hippocampus, olfactory bulb and SVZ, possibly due to the rich of NPCs in these places (Martín-Rivera et al., 1998; Caporaso et al., 2003; Zhou et al., 2011). Interestingly, ectopic telomerase in neurons and glial cells are also detected under special conditions (Iannilli et al., 2013). Elucidating the distribution of telomerase is important for understanding its specialized functions in the CNS.

### Expression of Telomerase in NSCs

During embryonic development, most of the cells in different tissues display telomerase expression and activities both in humans and rodents (Wright et al., 1996; Greenberg et al., 1998; Armstrong et al., 2005). High levels of telomerase activity have been observed throughout embryonic brain development (Fu et al., 2000). The activity level reaches a peak point at embryonic day 13 and then declines notably from embryonic day 13–18. The activity remains at a low level until postnatal day 3 when it decreases remarkably (Klapper et al., 2001; Mattson and Klapper, 2001). This expression pattern indicates that telomerase is present in the brain during embryonic development, which is supported by its elevated expression in embryonic neuronal stem or progenitor cells (Mattson and Klapper, 2001). Additionally, NSCs lose telomerase activity upon differentiating into astrocytes or neurons (Kruk et al., 1996; Miura et al., 2001; Caporaso et al., 2003; Cheng et al., 2007). It remains unclear how exactly telomerase activity decreases in differentiating cells. Sporadic research suggests that histone deacetylation and DNA methylation is involved in the silencing of the TERT gene, correlated with the decreased level of telomerase activity in differentiating cells (Lopatina et al., 2003; Hiyama and Hiyama, 2007; Würth et al., 2014).

Adult NSCs (ANSCs) populations are maintained during the adult lifetime in the SVZ and the SGZ in the lateral ventricle and the hippocampal DG (Gage, 2000; Arnold and Hagg, 2012). It is shown that telomerase activity contributes to the viability and self-renewal potential of ASCs including ANSCs (Ostenfeld et al., 2000; Allsopp et al., 2003; Liu et al., 2004; Choi et al., 2008). Indeed, telomerase activities and expression can be detected in the olfactory bulbs, SVZ and hippocampus (Caporaso et al., 2003).

Although the TERT level is significantly lower in the human brain compared to the mouse brain, it is still detectable (Horikawa et al., 2005). Remarkably, increased expression of human TERT is observed with activity in human neural progenitor cells (NPCs; Ostenfeld et al., 2000; Bai et al., 2004). Several human cells such as the teratocarcinoma NTera2 and human neuroblastoma (SK-N-SH) cell lines are generally used for investigating neuronal function (Jain et al., 2007). Abundant telomerase activity is enriched and inhibits neuronal differentiation in these cell lines (Jain et al., 2007; Richardson et al., 2007). Together, these studies display evidence of telomerase existence in both embryonic and adult NSCs.

### Ectopic Existence of Telomerase in Neurons and Glial Cells

Although it is believed that telomerase activity is restricted to areas containing stem cells in the brain (Caporaso et al., 2003), ectopic expression of the TERT protein has been shown in post-mitotic neurons without proliferating abilities (Iannilli et al., 2013). In contrast to the claim of Kang et al. (2004), that TERT is not detectable in the adult mouse brain using fluorescent *in situ* hybridization histochemistry, Spilsbury et al. (2015) presented evidence that TERT was expressed in cultured mouse neurons and microglia *in vitro*, which is consistent with a study by Fu et al. (2000). More interestingly, it is found that TERT was detected in the cytoplasm of mature human hippocampal neurons *in vivo* (Spilsbury et al., 2015). Additionally, TERT presents in activated microglia but is absent from astrocytes (Spilsbury et al., 2015). Various insults including ischemia, amyloid peptide administration, and glutamate or NMDA-induced excitotoxicity, substantially induce the expression of TERT in rodent neurons (Fu et al., 2000; Klapper et al., 2001; Zhu et al., 2001; Kang et al., 2004; Lee et al., 2010). Although telomerase levels are low in mature neurons, telomere repeat-binding factor 2 (TRF2) expression is high. Relative deficiency of TERT in new mature neurons during brain development may partially determine their vulnerability to DNA damage (Cheng et al., 2007). In addition, TERT is expressed in microglial cells in the hilus of hippocampus after administration of kainic acid in adult mice (Fu et al., 2002). The evidence of the existence of telomerase in neurons and glial cells implies a potential novel function in these cells, which warrants further investigation.

## Roles for Telomerase in Brain Development

Besides the roles of TERT in embryonic stem cells (ESCs), post-transcriptional regulation of TERT is implicated in the survival, self-renewal and differentiation of ASCs (Mattson et al., 2001; Marión and Blasco, 2010; Maeda et al., 2011; Cheng G. et al., 2013; Radan et al., 2014). This function is mediated by telomeric length stability or extra-telomeric telomerase isoforms (Radan et al., 2014; Zeng et al., 2014). In particular, telomerase deficiency impairs normal brain function in mice (Lee et al., 2010; Zhou et al., 2016, 2017). In the brain, telomerase in ASCs plays a critical role in the proliferation of NSCs, neuronal differentiation and development, and neuronal survival, which are involved in CNS diseases (Mattson and Klapper, 2001).

### Roles for Telomerase in Proliferation of NSCs

Telomerase is critical for stem cell proliferation. Using 3′-azido-2′,3′–dideoxythymidine (AZT), a type of telomerase activity inhibitor, Haïk et al. (2000) showed that telomerase activity was required for brain organogenesis. Additionally, cell proliferation of NPCs in the SGZ and olfactory bulb is severely decreased in the forebrain of TERC-knockout mice (Ferrón et al., 2009). We have also showed that AZT disrupted neurogenesis in the SGZ of the hippocampal DG both *in vivo* and *in vitro* (Zhou et al., 2011). In contrast, overexpression of telomerase by recombinant adenoviral vector expressing mouse TERT (Ad-mTERT-GFP) stimulates the proliferation of NSC both *in vitro* and *in vivo* (Zhou et al., 2011; Liu et al., 2012). Transduction with human TERT gene also results in increased proliferation in mouse NSCs (Smith et al., 2003).

### Roles for Telomerase in Neuronal Differentiation

NSCs possess the capability to self-renew and differentiate into mature nerve cells including neurons, astrocytes and oligodendrocytes (Miura et al., 2001; Ming and Song, 2011; Würth et al., 2014). The activity of telomerase rapidly decreases when NSCs stop dividing and differentiate into nerve cells (Kruk et al., 1996; Klapper et al., 2001). Therefore, the potential relationship between the decrease in telomerase activity and neuronal differentiation was examined. Indeed, overexpression of telomerase can inhibit neuronal differentiation in NPCs (Richardson et al., 2007). Inhibition of the telomerase activity by treatment of cells with telomerase antisense accelerates differentiation, suggesting that telomerase activity may contribute to the blockade of the onset of cell differentiation (Kondo et al., 1998). Moreover, overexpressing TERT in neuroepithelial precursors caused continuous cell division, but led to disaggregation and cell death, showing that TERT itself is not sufficient to cause termination of differentiation of neural precursors *in vitro* (Richardson et al., 2007). The telomere length regulated by telomerase activity may mediate the control of cell differentiation (Sharpless and DePinho, 2004).

However, a markedly different role for telomerase was reported in NCS differentiation. Schwob et al. (2008) demonstrated that overexpressing TERT in primary ESCs produced markers of neuronal precursors and mature neurons, with a heightened efficiency of neuroectodermal differentiation. It is also reported that TERT promotes neuronal survival and differentiation via reducing excitotoxicity in the CNS (Fu et al., 2002; Kang et al., 2004). Thus, telomerase activity and TERT expression may have different functions in regulation of cellular differentiation. A sharp reduction of telomerase activity during the development of the brain may be a useful signal for cells to begin the process of exiting the cell cycle, thereby differentiating into nerve cells including neurons and glial cells (Mattson et al., 2001). Studies have shown that decreasing telomerase activity was correlated with the differentiation of neural cell lines (Fu et al., 1999) including primary neurons (Fu et al., 2000), supporting such a mechanism. Telomerase-deficient mice reveal impaired neuronal differentiation, which is caused by the expression of RhoA effectors, Rock1 and Rock2, in parallel with the Notch pathway dependent on the modulation of p53 expression, supporting an opposing role of TERT in cell differentiation based on activity (Ferrón et al., 2009). In addition, abnormal telomerase expression significantly inhibited neuronal differentiation of NT2 cells, a model of human NPCs (Mattson et al., 2001). It is also unclear why only a subset of cells in neuronal-inducing conditions are able to attain or sustain terminal differentiation. Research investigating telomerase throughout human neuronal cell differentiation is needed to further answer these questions (Richardson et al., 2007). The different roles of telomerase expression and activity may be involved.

Although the roles of telomerase in neuronal differentiation are unsettled, it is certain that telomerase has important roles in the transition between pluripotent stem cells and committed neuronal cell fate in both NSC and ES cells (Schwob et al., 2008). Therefore, telomerase is a potential target for manipulating NSCs/NPCs, increasing the possibility for autologous cell replacement therapy for CNS illnesses including neurodegenerative diseases, psychiatric disorders, brain ischemia, aging and traumatic injury (Mattson et al., 2001; Sanai et al., 2005). More studies are necessary to determine the exact function of telomeres in neuronal differentiation.

### Roles for Telomerase in Neuronal Development

Brain-derived neurotrophic factor (BDNF) is an important molecule for neuronal development. It is found that telomerase is a key mediator of cell survival induced by BDNF in developing neurons (Fu et al., 2002). The high expression of TERT in neurons throughout embryonic and early postnatal development support an important role of telomerase in neuronal development (Mattson et al., 2001). Age-induced impairment of neurogenesis and neuritogenesis are correlated with the telomere length shortening in adult NPCs in the SVZ (Ferrón et al., 2009). The neurons matured from TERC-deficient NSCs fail to acquire a fully mature neuritic arbor (Ferrón et al., 2009). Our recent study found that TERT gene deletion caused a disruption in neuronal development, which was reversed by TERT reactivation (Zhou et al., 2017). The role of telomerase dysfunction in neuronal development may be involved in CNS diseases such as anxiety and memory deficiencies (Lee et al., 2010; Zhou et al., 2017).

### Roles for Telomerase in Neuronal Survival

Studies have provided evidence that telomerase may be involved in the regulation of the survival of cells including developing neurons (Fu et al., 2000; Klapper et al., 2001; Lu et al., 2001). Using antisense technology, Fu et al. (2000) demonstrated that suppression of TERT in cultured embryonic neurons induced apoptosis. The mechanisms by which TERT and/or telomerase are involved in survival-promoting activity may involve an interaction with neurotropic factors such as fiber growth factor (FGF), which can induce TERT expression (Haïk et al., 2000). Aside from FGF, Akt kinase, another neurotropic factor, affects TERT function. Phosphorylated TERT with enhanced enzymatic activity induced by Akt account for the neuronal survival-promoting actions of Akt (Mattson et al., 2001). Interestingly, temporary ectopic expression of TERT in neurons following brain ischemia protects hypoxic neurons from excitotoxicity, promoting neuronal survival (Kang et al., 2004; Li et al., 2011; Qu et al., 2011). In addition, TERT also mediates the neuronal survival-promoting actions of brain-derived neurotropic factor (BDNF), counteracting the adverse function of amyloid precursor protein in cultured hippocampal neurons during development (Zhu et al., 2001; Fu et al., 2002). Together, TERT activation is a common pathway for neurotropic factors including FGF, Akt, and BDNF, the well-established mediators of neuronal survival. Hence, the TERT subunit of telomerase in the embryonic brain may safeguard neuronal development, and the ectopic expression of TERT may act on post-mitotic cells to protect neurons from impairment.

Programmed cell death (apoptosis) is an important biological process (Shlezinger et al., 2017). Embryonic NSCs (ENSCs) play a critical role in the complex processes of the CNS formation during embryonic development and apoptosis of ENSCs contribute crucially to the appropriate formation of various biological structures and function of the brain (Gökhan and Mehler, 2001). The establishment of TERC knockout embryos led to a failure of closing the neural tube, which is crucially associated with telomere shortening (Herrera et al., 1999, 2000). TERC knockout mice also display a phenotype of enhanced apoptosis (Phelan et al., 1997). Consistently, reduction in TERT expression in ENSCs *in vitro* forces neurons to undergo apoptosis (Fu et al., 2000). These findings advocate both TERT and TERC as cell survival-promoting factors in neurons.

Most somatic cells enter a non-dividing state called cellular senescence after undergoing cell division (Wright and Shay, 1992). Cell cycle arrest frequently precedes activation of the molecular cascades of apoptosis, causing morphological changes and death in cells including neurons (Krantic et al., 2005). Interestingly, it is reported that telomerase activity cannot be detected and TERT expression is suppressed during growth arrest and cellular senescence (Wright et al., 1996). Once telomere length activates its checkpoint, cellular senescence is triggered, cell division is suspended and the cell eventually dies (Gilley et al., 2008). Since the well-established function of telomerase is maintaining the telomere length, it is reasonable to consider the involvement of telomerase in an anti-apoptotic role through prevention of DNA damage in a telomere-dependent manner (Rhyu, 1995; Liu, 1999). After differentiation from NSCs/NPCs, the somatic cells have extremely low levels of telomerase, leaving telomeres significantly damaged and cells vulnerable to stress, inducing apoptosis (Kondo et al., 1998). More direct evidence is shown in experiments suppressing TERT expression. In developing neurons, antisense-mediated silencing of TERT gene expression causes them to undergo apoptosis more frequently (Fu et al., 2000). In contrast, overexpression of TERT in human cells prevents cellular senescence and extends the lifespan (Bodnar et al., 1998; Yang et al., 1999). More importantly TERT can protect cultured neurons from apoptosis in experimental cell models relevant to ischemia and Alzheimer’s disease (AD; Zhu et al., 1999; Fu et al., 2000). It has been demonstrated that telomerase is associated with DNA repair and promotes cell survival (Peterson et al., 2001). One possible mechanism underling the role of TERT in apoptosis of NSCs is that TERT may suppress DNA damage and the activation of the associated pathways, which aids in the stabilization of chromosome ends (Holt et al., 1999). Collectively, both TERC and TERT may account for the action of telomerase in the modulation of apoptosis of NSCs at different stages, contributing to the balance of brain function.

## Telomerase and Diseases in the Central Nervous System

Since telomerase appears to have a significant role in the different stages of development of NSCs and NPCs, it may be involved in a variety of CNS diseases. Besides its well-studied role in brain cancer (Kheirollahi et al., 2013), telomerase also contributes to CNS impairment, including ischemic brain injury (Zhao et al., 2010; Li et al., 2011), neurodegenerative disease (Franco et al., 2006), mood disorders (Lee et al., 2010; Zhou et al., 2011) and schizophrenia (Kao et al., 2008; Porton et al., 2008).

### Brain Tumors

More than 85% of the tumor cells show telomerase activation for preventing progressive shortening of the telomere as excessive divisions (Harley, 1991). Tumors that originate in the brain are known as primary brain tumors, including astrocytomas, oligodendrogliomas and ependymomas. High telomerase activity was observed in astrocytoma including glioblastoma (GBM, grade IV astrocytoma), the most common type of malignant primary tumors in adults (Cheng L. et al., 2013). Hakin-Smith et al. (2003) found that alternative-lengthening-of-telomere is a prognostic indicator for patients with GBM (Lötsch et al., 2013). Consistently, Tchirkov et al. (2003) reported hTERT mRNA levels may represent a prognostic and diagnostic indicator for GBM patients. Mechanistically, two somatic mutations, C228T (–124 bp) and C250T (–146 bp) located upstream of the ATG start site confer enhanced TERT promoter activity in the GBM (Mosrati et al., 2015). These TERT promoter mutations are associated with shorter overall survival (Mosrati et al., 2015). In addition, the SNP of rs1006969, in the promoter regions, and rs2736100, in the intron 2, were reported to be associated with an increased risk of developing GBM (Mosrati et al., 2015). Interestingly, TERT promoter mutations led to a significant increase in TERT mRNA and enhanced activity of telomerase in tumors. Based on these findings, Marión and Blasco (2010) used a telomerase antagonist, imetelstat, to target glioblastoma tumor-initiating cells efficiently for decreasing proliferation and tumor growth (Ferrandon et al., 2015). However, using telomerase-based drugs for cancer treatment should be conducted with caution, as these therapies may have adverse effects normal tissues.

Following the findings of elevated levels of telomerase in tumors, new anticancer methods targeting telomerase have been highly anticipated. In 1995, the first attempt to use an antisense vector against TERC was reported (Feng et al., 1995). To date, a number of different approaches including antisense, natural compounds, hormones, vaccines, and small molecules have been developed to inhibit telomerase activity in cancer cells (Saretzki, 2003; Shay and Wright, 2006). However, no company has declared success in developing compounds for cancer treatment. Several factors account for this lack of progress. It takes a long time for telomerase inhibition to be clinically effective, which may cause toxicity in normal proliferative cells (Harley, 2008). Another factor is the pharmaceutical industry arguing that telomerase is not a practical drug target (Man et al., 2016). Many efforts are being made to continue investigating novel methods, including oncolytic viral strategies and immunotherapy, to target telomerase (Olaussen et al., 2006). Despite significant progress, issues must be addressed before applying telomerase-based therapies for treating cancer including brain tumors.

### Aging Brain

Telomeres play a central role in aging. Shortening of telomeres has been linked to the mechanisms responsible for the aging of cells (López-Otín et al., 2013). Telomerase, preventing the telomere from being too short, thus acts as an anti-aging enzyme, proposing a “telomere theory of aging”, a prominent concept in research (Jaskelioff et al., 2011). Since the secret of “the end replication problem” was uncovered owing to the finding of telomerase, its role in cellular aging was predicted and revealed (Greider and Blackburn, 2004). TERT gene knockout studies provide direct evidence that TERT loss provoked tissue degeneration including progressive atrophy of tissues, depletion of stem cells, failure of organ systems and impairment of tissue response to injuries throughout the whole body including the brain (Jaskelioff et al., 2011). Strikingly, 4 weeks of reactivating telomerase reversed the aging process in the brain, including NSCs proliferation and differentiation, brain size and olfactory function (Jaskelioff et al., 2011). Overexpression of TERT in NSCs or neurons is beneficial for the adult brain by increasing resistance to neurodegenerative changes with aging (Mattson et al., 2001). Clinical evidence also suggests a correlation between telomerase activity in human leukocytes and the volume of the hippocampus in early stages of aging (Jacobs et al., 2014). As noted, the aging brain is associated with extensive accumulation of DNA damage. Telomerase gene therapy in adult and old mice has been shown to delay aging and increase longevity without causing cancer via DNA damage repair (Bernardes de Jesus et al., 2012). Thus, telomerase could be a serious intervention to inhibit degeneration in the aging brain.

AD, with two discrete pathologies including Amyloid-β (Aβ) and tau (p-tau) aggregation, is a common neurodegenerative disorder in elderly patients, and has aging characteristics featured with cell senescence and oxidative stress (Smith et al., 1995; Spilsbury et al., 2015). It is shown that the length of neuronal telomeres are remarkably shorter in hippocampal neurons in patients with AD (Franco et al., 2006). TERT exhibits neuronal protective properties against tau pathology in experimental models of AD (Kota et al., 2015; Spilsbury et al., 2015). Additionally, ROS generation and oxidative damage in neurons, the mediators of pathological tau, are relieved in TERT knockout mice (Spilsbury et al., 2015). In accordance with this evidence, Aβ oligomers-induced cytotoxicity is shown to be potentially mediated by telomerase activity inhibition (Wang et al., 2015). However, TERC knockout mice with AD present with telomere shortening which slows down the progression of Aβ pathology (Rolyan et al., 2011). The inconsistency of telomere in AD pathology may be due to different processes, provoking microglial activation with extreme telomere shortening (Rolyan et al., 2011). Therefore, telomerase may play different roles in the tau and amyloid pathology via multiple mechanisms. Furthermore, leukocyte telomere length is altered in other neurodegenerative disorders including Huntington’s disease and dementia, indicating that change in telomere length is a shared characteristic of neurodegenerative disorders (Kota et al., 2015). Thus, the modification of telomerase together with telomere could be a marker of aging-related conditions (López-Otín et al., 2013).

### Parkinson’s Disease

Parkinson’s disease (PD) is an aging-associated long-term degenerative disorder (Singleton and Hardy, 2016). Contrary to short telomeres observed in the aging brain, the relationship between telomere and PD remains unclear. An analysis of 131 PD patients and 115 healthy controls performed by Eerola et al. (2010) found no difference in telomere length between PD patients and healthy controls. Consistently, a case-control study from Wang et al. (2008) reported that shorter telomeres are not associated with a higher risk of PD. Moreover, a large nested case-control study also found that telomere shortening was associated with reduced PD risk (Schürks et al., 2014). To date there is no evidence showing abnormalities of telomerase or telomere in the CNS tissues of PD patients.

### Amyotrophic Lateral Sclerosis

Amyotrophic lateral sclerosis (ALS) is another type of progressive neurodegenerative disease, characterized by the death and dysfunction of nerve cells in the CNS (Boillée et al., 2006; Eitan et al., 2012). Motor neurons in the spinal cord, the cerebral cortex, and brain stem gradually break down and die, causing muscle weakness and atrophy. An investigation on the telomere length in blood leukocytes revealed accelerated telomere attrition and lower levels of telomerase in patients with ALS (De Felice et al., 2014). More importantly, human TERT expression in the spinal cord of ALS patients is extremely low compared to healthy controls (De Felice et al., 2014). A controlled and transient increase in telomerase expression and activity in the brain using a telomerase-increasing compound delayed the onset and progression of ALS (Eitan et al., 2012). This compound increased the survival of motor neurons in the spinal cord by 60% (Eitan et al., 2012). Telomerase-related DNA repair and transcription regulation may contribute to the survival of motor neurons in ALS (Singh et al., 2017). Surprisingly, Linkus et al. (2016) found a trend of longer telomeres in microglia from human post-mortem brain tissue with ALS. However, knocking out telomerase in mice accelerated the ALS phenotype (Linkus et al., 2016). The longer telomeres in microglial cells may play a role in microglial proliferation, which contributes to ALS disease progression (Linkus et al., 2016). Therefore, the contribution of telomerase in different neural cells in the development of ALS may have distinct mechanisms.

### Brain Ischemia

Normally, TERT expression and telomerase activity are at a very low level and undetectable in post-mitotic cells including neurons in the brain. After ischemic injury, ectopic expression of TERT was detected in neurons (Kang et al., 2004). Transgenic overexpression of TERT showed a significant resistance to injury. Induction of TERT in injured neurons protects against NMDA excitotoxicity, ameliorating ischemic neuronal cell death (Kang et al., 2004). Aside from neurons, astrocytes appear to have a role in TERT-related neuronal protection. Baek et al. (2004) show TERT co-localization with glial fibrillary acidic protein (GFAP), a marker of astrocyte, in the neonatal brain 3 days after stroke. Consistently, it was reported that TERT mRNA and protein were up-regulated in neurons 2 days after hypoxia-ischemia but shifted to astrocytes at day 3 (Qu et al., 2011). The distribution of temporary ectopic expression of TERT supports the concept that both promotion of neuronal survival and attenuation of astrocyte proliferation in the developing brain contribute to a TERT-based neuroprotective mechanism of hypoxia–ischemia (Qu et al., 2011). Additionally, a shift of the cytosolic free Ca^2+^ into the mitochondria is important for TERT to inhibit apoptosis and excitotoxicity, decrease angiogenesis and promote neuronal survival (Li et al., 2011). Reduction of telomerase activity leads to an intensified neuroinflammatory response and blood-brain barrier disruption after experimental stroke (Zhang et al., 2010). Decreasing ROS generation and increasing mitochondrial membrane potential were also reported as TERT’s neuroprotective mechanisms (Li et al., 2013). Better understanding these novel mechanisms may assist in the development of more effective neuroprotective strategies in the treatment of ischemic brain injury.

### Mood Disorders

The World Health Organization ranks mood disorders as the leading causes of years (Murray and Lopez, 1996). Brain structural and functional abnormalities mediate the pathophysiology of mood disorders, including major depressive disorder (MDD), bipolar disorder (BD), and anxiety. Increasing studies suggest a strong causal link between impaired neurogenesis and etiology of mood illnesses. Considering the function of telomerase in stem cells, especially ANSCs/ANPCs, it is highly expected that telomerase plays an important role in the modulation of mood disorders (Monroy-Jaramillo et al., 2017). Epidemiologic studies reveal a close association between telomere length and psychiatry illness (Darrow et al., 2016). Dysfunctional telomeres in peripheral leukocytes have been observed in several psychiatric conditions (O’Donovan et al., 2011; Lindqvist et al., 2015). A pilot study found that 16 un-medicated subjects with MDD had increased basal telomerase activity in comparison with healthy controls in males (Wolkowitz et al., 2012) but not females (Simon et al., 2015). Post-mortem research shows a significant reduction in telomere length across brain regions, especially in the hippocampus, of patients with MDD (Mamdani et al., 2016). Repression of telomere-associated genes leads to microglial senescence, a mechanism of neuropsychiatric diseases (Kronenberg et al., 2017).

Life stress is the main environmental factor causing MDD. Accelerated telomere shortening and decreased telomerase activity has also been reported in response to chronic stress (Epel et al., 2004, 2010). Chronic mild stress lowered hippocampal TERT protein levels and telomerase activity was reversed by fluoxetine treatment (Zhou et al., 2011). In addition, a low level of TERT and its activity was detected in the hippocampus in a rat model of MDD (Wei et al., 2015). Both overexpression of TERT and TERT activity inhibition or knockout demonstrated that hippocampal telomerase played an essential role in modulating depressive and aggressive behaviors (Zhou et al., 2011, 2016).

Deficiencies in telomerase can also be associated with other mood disorders. Telomerase expression is correlated with anxiety (Perna et al., 2016). Brain structural and functional changes of aging were more pronounced in subjects with anxiety than controls, including reduced gray matter density, white matter alterations, and impaired functional connectivity of large-scale brain networks. Moreover, molecular correlates of brain aging such as telomere shortening, Aβ accumulations, and oxidative/nitrosative stress, were overrepresented in anxious subjects (Perna et al., 2016). Both rodent and human findings showed an association between anxiety and telomere shortening. TERT-deficient mice displayed significantly higher anxiety-like behaviors (Lee et al., 2010). Consistently, deficiency of telomerase resulted in increased anxiety-like behavior in aged transgenic mice (Lee et al., 2011). In non-psychiatric human brain samples, associations were found between exposure to chronic stress (e.g., childhood adverse experiences/stressful caregiving status) or high phobic anxiety and accelerated telomere shortening, which may be related to dysregulation of inflammatory markers, HPA axis, and autonomic system function (Surtees et al., 2011; Okereke et al., 2012; Wolkowitz et al., 2012; Révész et al., 2014).

### Schizophrenia

Generally, schizophrenia is not regarded as an aging-related disorder. However, pathology of aging may be a component of this disorder, since there are similar structural brain abnormalities (Buchsbaum and Hazlett, 1997; DeLisi, 1997; Surtees et al., 2011; Okereke et al., 2012; Wolkowitz et al., 2012; Révész et al., 2014). Research shows the average length of telomeres in peripheral blood lymphocytes from individuals with schizophrenia is markedly shortened (Kao et al., 2008; López-Otín et al., 2013). A recent study also demonstrated shorter telomere length among patients with schizophrenia (Galletly et al., 2017). In line with this finding, a significant decrease was reported in telomerase activity in peripheral blood lymphocytes taken from individuals with schizophrenia (Porton et al., 2008). While these studies provide exciting correlative results, further research is needed to determine the exact role of telomerase in schizophrenia.

### Summary

More and more evidence displays a correlation between changes in TERT activity or telomere length and CNS diseases besides brain tumors. Therefore, it is possible that additional applications of TERT manipulation may be useful in the treatment of various CNS disorders. Due to potential adverse effects on normal cells, treatments based on TERT manipulation must be carefully planned and should exclude patients with conditions such as tumorigenesis. Regardless, this is an exciting new avenue for research and translational medicine, as scientists are making breakthroughs in telomerase gene therapy (Bernardes de Jesus et al., 2012). Bernardes de Jesus et al. (2012) show that administration of TERT to aged mice, using an adeno-associated virus, reduced the incidence of glucose intolerance and osteoporosis, and improved the function of the neuromuscular junction. More notably, they found this gene therapy strategy improved the ability of memory formation without increasing tumorigenesis (Bernardes de Jesus et al., 2012). While these new studies are encouraging, there is still much research needed before telomerase therapy can be translated into clinical application for the treatment of CNS disorders.

## Conclusion and Perspectives

In this review article, we have presented the existence of telomerase in the CNS and its roles in the developing and adult brain, including proliferation, differentiation, maturation and apoptosis of nerve cells, as well as CNS diseases. The mechanisms whereby telomerase acts within these processes are becoming more clear, however its exact role and mechanisms remain unknown (Harley, 1991). Small molecules screened and tested for telomerase inhibition targeting telomerase activity are anticipated for drug discovery of cancers, although they are not yet approved (Harley, 2008). Understanding the precise functions of TERT and other telomere-associated proteins in the CNS may be helpful for recruiting them as novel targets for treatment of brain tumor, neurodegenerative diseases, and mood disorders. Hence, knowing the role of telomerase in the nervous system is not only important to gain further insight into the process of the neural cell cycle, but also provides a novel therapeutic application for the treatment of nervous system diseases. Although failure of developing telomerase therapeutics for clinical use is possible, the manipulation of the telomere/telomerase system is still a promising and novel approach for therapeutic purpose. The knowledge of this system, reviewed here, may be vital in the development of future treatments of neurological dysfunction.

## Author Contributions

Q-GZ and M-YL wrote the manuscript. AN edited the manuscript.

## Conflict of Interest Statement

The authors declare that the research was conducted in the absence of any commercial or financial relationships that could be construed as a potential conflict of interest.
